# Olfml3 Regulates Microglial Inflammation and Neuronal Injury in Obstructive Sleep Apnea via Cybb‐Mediated TLR4/NF‐κB Pathway

**DOI:** 10.1002/cns.71006

**Published:** 2026-07-08

**Authors:** Deqiu Kong, Yaowen Wang, Xianjun Chen, Cihao Hu, Bin Zhang, Xing Chen

**Affiliations:** ^1^ Otorhinolaryngology‐Head and Neck Surgery The First Affiliated Hospital of Ningbo University Zhejiang China

**Keywords:** Cybb, microglia, Olfml3, sleep apnea syndrome, TLR4/NF‐κB

## Abstract

**Background:**

Neurocognitive impairment in obstructive sleep apnea (OSA) is primarily driven by intermittent hypoxia (IH)‐triggered neuroinflammation, where microglia play a pivotal role. The involvement of Olfml3 in IH‐induced neuroinflammation remains unclear.

**Methods:**

Single‐cell RNA sequencing (scRNA‐seq) data from the hippocampi of IH‐induced OSA mice were analyzed to identify cell subpopulations, with further focus on Olfml3's differential expression, enriched pathways, and differentiation trajectories in microglia. An in vitro OSA model was established using IH‐treated microglia. qRT‐PCR and western blot (WB) were utilized to assess Olfml3 and cytochrome b (Cybb) expression. Microglial polarization was evaluated via flow cytometry, while Enzyme‐Linked Immunosorbent Assay (ELISA) was applied to quantify inflammatory cytokines. Reactive oxygen species (ROS) were detected using fluorescent probes, and TLR4/NF‐κB pathway activation was verified by WB assessment of Toll‐like receptor 4 (TLR4), phosphorylated‐p65 (p‐p65), and p65 expression. Neuronal injury was assessed by treating neurons with microglial‐conditioned medium, followed by CCK‐8 for viability assessment and flow cytometry for apoptosis analysis. An in vivo OSA model was constructed by exposing mice to IH treatment. Cognitive deficits of mice were evaluated using the Morris water maze and blood oxygen saturation measurement, while pathological changes in brain tissue and cell apoptosis were examined via HE and TUNEL staining. Immunohistochemistry staining was employed to detect Olfml3 and Cybb protein levels. An immunofluorescence assay was conducted to measure Iba1 for evaluating microglial activation. ROS levels were detected by using fluorescent probes. The expression of TLR4/NF‐κB pathway proteins was assessed by WB. CD86/CD206 ratios were analyzed by flow cytometry, and the expression of inflammatory cytokines was analyzed by ELISA.

**Results:**

scRNA‐seq revealed reduced microglial proportions under hypoxia, with further analysis revealing that Olfml3 in microglia had a negative correlation with Cybb. An IH‐induced OSA model confirmed that Olfml3 overexpression alleviated microglial inflammation and neuronal injury by suppressing the TLR4/NF‐κB pathway via Cybb. In vivo experiments further validated Olfml3's protective role against IH‐induced neuroinflammation in OSA.

**Conclusion:**

Olfml3 in microglia mitigates IH‐induced proinflammatory activation and neuronal injury via the Cybb/TLR4/NF‐κB axis, thereby conferring neuroprotection against OSA‐associated neuroinflammation.

## Graphical abstract

In obstructive sleep apnea (OSA), intermittent hypoxia (IH) induces downregulation of Olfml3 in microglial cells and inhibits the activation of microglial cells; Low Olfml3 expression leads to increased expression of the downstream gene Cybb; Cybb activates the TLR4/NF‐κB inflammatory pathway, driving microglial M1 polarization and the release of proinflammatory cytokines, ultimately resulting in neuronal damage.

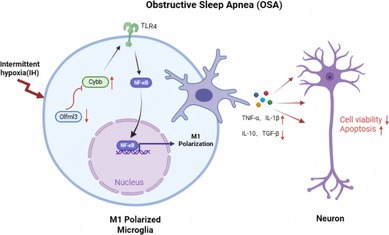

## 1 Introduction

Obstructive sleep apnea (OSA), the most prevalent sleep‐related breathing disorder, is characterized by recurrent upper airway collapse during sleep, leading to intermittent hypoxia (IH) [1]. IH induces neuroinflammation and neuronal apoptosis in the central nervous system (CNS), resulting in behavioral and functional deficits [2]. Mounting evidence implicates microglia, the resident immune cells of the CNS, in the initiation and perpetuation of neuroinflammation in OSA patients [3]. Microglia polarize into proinflammatory (M1) or anti‐inflammatory (M2) phenotypes in response to microenvironmental changes [4]. Under IH, M1 polarization of microglia promotes proinflammatory cytokine release, exacerbating neuronal dysfunction and neuroinflammation [5, 6]. Thus, targeting microglial polarization is a promising therapeutic strategy for IH‐induced neuroinflammation in OSA. Exploring the key functions of microglia in OSA is of great significance for developing new therapeutic targets.

Notably, neuroinflammation in CNS diseases hinges on inflammatory pathway activation. Notch, p38‐MAPK, and Wnt/β‐catenin pathways have been implicated in microglial inflammation [7, 8, 9]. Specifically, IH activates pattern recognition receptors (e.g., Toll‐like receptor 4, TLR4) on microglia. TLR4, expressed in neurons, microglia, and astrocytes, triggers nuclear factor κB (NF‐κB) signaling to amplify inflammatory mediator release [10, 11]. TLR4/NF‐κB activation is strongly associated with microglial inflammation [10, 12], making it a critical therapeutic target. However, the mechanisms underlying TLR4/NF‐κB activation in OSA remain elusive.

Olfactomedin‐like 3 (Olfml3), a glycoprotein of the olfactomedin‐domain family, regulates neurodevelopment and immune responses [13]. It mediates cell adhesion, signaling, and extracellular matrix interactions, playing key roles in tissue development and intercellular communication [14]. Notably, Olfml3 is highly expressed in the brain, suggesting its important role in neurodevelopment and neurological function [15]. However, despite emerging insights into its functions in neurodevelopment and immune regulation, the involvement of Olfml3 in neurological complications associated with OSA remains unexplored. Chronic intermittent hypoxia resulting from OSA triggers neuroinflammation and microglial activation within the central nervous system, ultimately leading to neuronal injury. Given the putative immunomodulatory and tissue‐repair functions of Olfml3 [16], we hypothesized that it may critically regulate microglia‐mediated inflammatory responses and neuronal survival under OSA‐related pathological conditions. Accordingly, this study aimed to elucidate the specific effects of Olfml3 on microglial inflammation and neuronal injury in the context of OSA, with the goal of establishing a theoretical foundation for its potential as a therapeutic target for OSA‐associated neurological complications.

Herein, we constructed a single‐cell atlas of OSA via single‐cell RNA sequencing (scRNA‐seq), delineating cellular subpopulations and Olfml3's microglial functions. In vitro and in vivo studies demonstrated that Olfml3 inhibits the TLR4/NF‐κB pathway by downregulating Cytochrome b (Cybb). The discovery of this mechanism revealed the potential role of Olfml3 in alleviating the inflammation of microglia and neuronal injury associated with OSA, providing an important theoretical basis for the therapeutic intervention of neuroinflammation related to OSA.

## 2 Methods

### 2.1 scRNA‐Seq Data Source and Quality Control

The scRNA‐seq data were extracted from the Sequence Read Archive (SRA) database (PRJNA1089719), with one normoxic and one hypoxic mouse hippocampal sample. The “Seurat” package in R software was used for downstream analysis. Thresholds were applied to exclude low‐quality cells and low‐expression genes [1]: genes per cell are between 200 and 8000 [2]; unique molecular identifiers per cell are between 300 and 20,000 [3]; mitochondrial gene expression per cell is less than 5%.

### 2.2 scRNA‐Seq Analysis

The NormalizeData function was used to normalize the dataset, and 2000 highly variable genes were identified. Then, the data were scaled by the ScaleData function, and principal component analysis was performed. The results were visualized by the RunUMAP function. During downstream analysis, the “Harmony” package was used for batch correction to prevent batch effects from interfering with the analyses. Subsequently, the FindClusters function was used for cell clustering. In the end, we annotated cell populations in OSA according to previous studies and identified differentially expressed genes among each cell type using the FindAllMarkers function in the “Seurat” package.

### 2.3 GO And KEGG Enrichment Analyses

The “ClusterProfiler” package was used for Gene Ontology (GO) and Kyoto Encyclopedia of Genes and Genomes (KEGG) enrichment analyses of genes and visualization.

### 2.4 Pseudotime Analysis

Pseudotime trajectory analysis was performed using Monocle (version 2.26.0). All data from the Seurat object were imported into a Monocle CellDataSet (CDS) object. Dimensionality reduction was then performed with the DDRTree algorithm, and the resultant trajectory was visualized using default parameters.

### 2.5 Animal Model Construction

Animal experiments were approved by the Animal Ethics and Use Committee of the Ethics Committee for Animal Experiments of Guangdong Medical Laboratory Animal Center Institution, Approval Number D202506‐14. Thirty specific pathogen‐free (SPF) male C57BL/6 mice, aged 6 to 8 weeks, were obtained from Model Organisms (China) and randomly assigned to five groups. Mice in the control group (*n* = 6) were maintained under standard housing conditions, whereas those in the remaining four groups were subjected to IH to establish an animal model of OSA.

Lentivirus preparation and administration: Oligonucleotides targeting Olfml3 or Cybb mRNA were designed and inserted into the pLVX‐Puro lentiviral vector. The resultant constructs were co‐transfected into HEK293T cells together with the packaging plasmids psPAX2 and pMD2.G to generate lentiviral particles. Following concentration and purification, a lentiviral suspension with a titer of 1 × 10^9^ TU/mL was obtained. One week prior to OSA modeling, lentiviral delivery was performed according to group assignments (oe‐NC + oe‐NC, oe‐Olfml3 + oe‐NC, and oe‐Olfml3 + oe‐Cybb; *n* = 6 per group). Briefly, mice were anesthetized with isoflurane and secured in a stereotaxic frame. Injection sites in the hippocampus were selected based on a mouse brain atlas (anteroposterior: −2.0 mm from bregma; mediolateral: ±1.5 mm from the midline; dorsoventral: −1.5 mm from the dura). A volume of 1 μL of the lentiviral suspension was slowly infused into each side of the hippocampus at a rate of 0.2 μL/min using a microinjection pump. Following the injection, the needle was left in place for 5 min to allow adequate diffusion.

The IH procedure was started by introducing N_2_ to reduce oxygen concentration in the chamber to 5% under 2 min, followed by O_2_ administration to restore normoxia to 22% within another 2 min. For 6 weeks, mice in the IH group received 8 h of daily IH treatment, with the remaining 16 h spent under normoxic conditions identical to the normoxic control group. When the Morris water maze (MWM) test was completed, mice were euthanized using sodium pentobarbital (MCE, USA). Mouse hippocampal tissues were collected for later experiments.

### 2.6 Cell Culture

Mouse microglial cells BV2 (BNCC337749, BNCC, China) and mouse hippocampal neuronal cells HT22 (BNCC358041, BNCC, China) were cultured in DMEM‐H complete medium (Thermo Fisher, USA) with 10% fetal bovine serum (Beyotime, China) and 1% penicillin–streptomycin (Beyotime, China) at 37°C with 5% CO_2_.

The supernatant of BV2 cell cultures was collected and mixed with an equal volume of complete medium to prepare conditioned medium (CM). HT22 cells were then cultured with the CM at 37°C with 5% CO_2_ for 24 h. The HT22 cells were collected for subsequent experiments.

### 2.7 IH Cell Model Construction

BV2 microglial cells were subjected to IH in a tri‐gas incubator equipped with precise gas concentration control to establish an IH cell model. Hypoxia–reoxygenation cycles were achieved by continuously introducing high‐purity N_2_ and CO_2_ into the chamber, with real‐time oxygen concentration monitoring. To maintain a stable medium pH, the CO_2_ concentration was kept constant at 5% throughout the procedure. The IH cycling parameters were as follows: Each cycle lasted 30 min, during which N_2_ was first infused to displace ambient air, reducing the O_2_ concentration to 0% within 15 min (hypoxia), followed by the introduction of compressed air to restore O_2_ to 22% ± 0.1% within the next 15 min (reoxygenation). Following 12 h of IH exposure, BV2 cells were harvested for subsequent experiments. Concurrently, cells in the normoxic control group were cultured under static conditions in a standard incubator at 22% O_2_ and 5% CO_2_ for an equivalent duration. To rule out nonspecific cell death due to extreme conditions, cell viability was verified in preliminary experiments using the CCK‐8 assay.

### 2.8 Cell Transfection

Overexpression plasmids (oe‐Cybb, oe‐Olfml3) and their corresponding negative controls (oe‐NC), as well as small interfering RNA targeting Olfml3 (si‐Olfml3) and its negative control (si‐NC), were obtained from RiboBio (China). Relevant sequence information is provided in Supporting Information 1.

For transfection, BV2 microglial cells were seeded at an appropriate density in DMEM complete medium without antibiotics 24 h prior to the procedure. When cell confluence reached 70%–90%, transfection was carried out using Lipofectamine 2000 (Thermo Fisher, USA). Briefly, Lipofectamine 3000 was diluted in Opti‐MEM medium (Gibco, USA) separately from either plasmid DNA or siRNA, after which the two diluted solutions were combined at a 1:1 volume ratio and allowed to incubate at room temperature for 20 min to form transfection complexes. The resulting mixture was then added to the culture wells and gently mixed. Following a 6 h incubation at 37°C, the medium was replaced with complete DMEM containing 10% fetal bovine serum to minimize potential cytotoxicity associated with the transfection reagent. At 48 h post‐transfection, cells were collected, and transfection efficiency was assessed by visualizing GFP‐positive cells under a fluorescence microscope (Figure S2). Only batches meeting quality criteria were used for subsequent experimental analyses.

### 2.9 qRT‐PCR

The reverse transcription started with extracting total RNA using TRIzol reagent from Invitrogen, USA, and was performed using a reverse transcription kit from the same institution to transcribe RNA into cDNA. qRT‐PCR analysis was then performed using primers and SYBR Green Master Mix (Vazyme, China) in the real‐time PCR system (Bio‐Rad, USA). The expression of Olfml3 and Cybb was quantified by applying the 2^−ΔΔCT^ method with the internal reference gene GAPDH. Gene‐specific primers were as follows: Olfml3: F: AGCTGCCTTAGAGGAACGG, R: CCTCCCTTTCAAGACGGTCC; Cybb: F: TCCTATGTTCCTGTACCTTTGTG, R: GTCCCACCTCCATCTTGAATC; GAPDH: F: CATCACTGCCACCCAGAAGACTG, R: ATGCCAGTGAGCTTCCCGTTCAG.

### 2.10 Western Blot (WB)

The research used RIPA buffer (Beyotime, China) to lyse cells and collect total protein, whose supernatant was then mixed with sodium dodecyl sulfate polyacrylamide gel electrophoresis (SDS‐PAGE) protein loading buffer (Beyotime, China) and boiled for denaturation. Proteins were later separated by electrophoresis and transferred to a polyvinylidene fluoride (PVDF) membrane (Beyotime, China). After blocking with 5% skim milk for 1 h, the membrane was incubated with primary antibodies at 4°C overnight, and with secondary antibodies at room temperature for 1 h afterward. Protein bands were detected by chemiluminescence (Millipore, USA), and gray values of the bands were analyzed using ImageJ (NIH, USA). Antibodies used were as follows: Rabbit anti‐Olfml3 antibody (PA5‐31581, Thermo Fisher, USA) at a dilution of 1:3000; rabbit anti‐Cybb antibody (PA5‐79118, Thermo Fisher, USA) at a dilution of 1:1000; rabbit anti‐TLR4 antibody (48–2300, Thermo Fisher, USA) at a dilution of 1:200; rabbit anti‐p‐NF‐κB p65 antibody (ab239882, Abcam, UK) at a dilution of 1:1000; rabbit anti‐NF‐κB p65 antibody (ab207297, Abcam, UK) at a dilution of 1:30; rabbit anti‐GAPDH antibody (ab181602, Abcam, UK) at a dilution of 1:60; goat anti‐rabbit IgG H&L (HRP) antibody (ab6721, Abcam, UK) at a dilution of 1:5000.

### 2.11 Immunofluorescence (IF)

BV2 cells and brain tissues were fixed with 4% paraformaldehyde (Beyotime, China) at room temperature for 15 min and washed three times with PBS. The samples were then permeabilized with 0.3% Triton X‐100 (Beyotime, China) for 10 min and blocked with 5% bovine serum albumin (BSA) (Beyotime, China) for 30 min. Subsequently, the samples were incubated with ionized calcium‐binding adapter molecule 1 (Iba1) antibody (ab178846, Abcam, UK) at a dilution of 1:1200, at 4°C overnight. After washing, the samples were incubated with fluorophore‐conjugated secondary antibody (ab150078, Abcam, UK) at a dilution of 1:1000, at 37°C for 1 h, and then counterstained with DAPI (Beyotime, China) for 5 min. Fluorescence images were captured using a fluorescence microscope (Olympus, Japan).

### 2.12 Immunohistochemistry (IHC)

Mouse brain tissues were fixed in 4% paraformaldehyde (Beyotime, China), embedded in paraffin, and cut into 4 μm‐thick sections. After deparaffinization with xylene and rehydration, citrate buffer was used for antigen retrieval (Beyotime, China). The sections were then incubated with Olfml3 antibody (PA5‐31581, Thermo Fisher, USA) at a dilution of 1:1000, and Cybb antibody (PA5‐79118, Thermo Fisher, USA) at a dilution of 1:200, at 4°C overnight, respectively, followed by incubation with secondary antibody (ab6721, Abcam, UK) at a dilution of 1:50, at 37°C for 30 min. Sections were firstly stained using a DAB kit (Solarbio, China), then counterstained with hematoxylin (Beyotime, China), and mounted with neutral resin. Finally, the sections were observed under a microscope (Olympus, Japan).

### 2.13 MWM Test

In the MWM test, spatial learning and memory of the mice were evaluated. The apparatus consisted of a circular pool (130 cm in diameter, 60 cm in height) filled with opaque water maintained at 22°C ± 2°C, and a camera connected to the video tracking system. A round escape platform was placed 1 cm below the water surface. Mice were randomly placed in the water facing the pool wall from one of four quadrants and given 60 s to locate the platform. This procedure was repeated three times daily for five consecutive days. Mice who failed to find the platform within 90 s were guided to it and stayed for 15 s. On the sixth day, the platform was removed. Mice were allowed to freely swim in the pool for a 60‐s probe test, during which the percentage of time spent in the target quadrant, the time of crossing the platform, and the swimming path were recorded.

### 2.14 Enzyme‐Linked Immunosorbent Assay (ELISA)

The levels of tumor necrosis factor (TNF)‐α, interleukin (IL)‐1β, IL‐10, and transforming growth factor (TGF)‐β in cells and tissues were measured using ELISA kits (Elabscience, China) according to instructions. The absorbance at 450 nm was measured using a microplate reader (Thermo Fisher, USA), and the concentrations were calculated based on standard curves.

### 2.15 Cell Counting Kit‐8 Assay

Cells were seeded at 2 × 10^3^ cells/well in 96‐well plates. After IH treatment and 24 h of culture, each well was added with 10 μL of CCK‐8 reagent (Beyotime, China), and incubated at 37°C for 4 h. The absorbance at 450 nm was measured using a microplate reader (Thermo Fisher, USA).

### 2.16 Flow Cytometry

Flow cytometry was used to analyze the polarization of BV2 cells (M1/M2). Cells were first resuspended in 100 μL PBS and stained with anti‐CD206‐PE (12–2061‐82, Thermo Fisher, USA) and anti‐CD86‐APC (17–0862‐82, Thermo Fisher, USA) antibodies at 4°C in the dark for 20 min. After cleaning with PBS, the cells were analyzed using a FACSCalibur flow cytometer (BD Bio, USA).

To detect HT22 cell apoptosis, cells were collected and washed twice with PBS, then stained with Annexin V‐FITC and propidium iodide (BD Bio, USA) at room temperature in the dark for 30 min. After PBS washing, the cells were analyzed using a FACSCalibur flow cytometer (BD Bio, USA).

### 2.17 Reactive Oxygen Species (ROS) Detection

ROS production was measured using a ROS assay kit (Beyotime, China). Following instructions, ROS levels within cells and tissues were quantified using the DCFH‐DA fluorescence probe and a FACSCalibur flow cytometer (BD Bio, USA).

### 2.18 Blood Oxygen Saturation (SpO_2_ ) Measurement

Regional cerebral SpO_2_ was assessed using a Moor vascular monitoring system combined with a laser Doppler perfusion and temperature monitor (Moor Instruments Ltd., UK). Mice were anesthetized with 5% isoflurane and placed in a prone position for immobilization. Following disinfection of the midline parietal region, a longitudinal incision was made to fully expose the skull. The detection probe was positioned 2 mm posterior to the bregma and 4 mm lateral to the midline, with data acquisition conducted over 15 s per site. Cerebral SpO_2_ readings were obtained via laser Doppler technology.

### 2.19 Hematoxylin and Eosin (HE) Staining

Paraffin‐embedded brain tissue sections were deparaffinized with xylene and rehydrated through a graded ethanol series (100%, 95%, 85%, 70%, and 50%). The sections were stained with hematoxylin (Beyotime, China) for 5 min, differentiated in hydrochloric acid ethanol, then rinsed for 2 min, and counterstained with eosin (Beyotime, China) for 10 to 15 s. After dehydration and clearing with xylene, the sections were mounted with neutral resin and observed under a microscope (Olympus, Japan).

### 2.20 TdT‐Mediated dUTP Nick‐End Labeling (TUNEL) Staining

After deparaffinization and antigen retrieval, brain tissue sections were treated with 0.3% Triton X‐100 (Beyotime, China) for 5 min and incubated with TUNEL reagent (Beyotime, China) at 37°C in the dark for 1 h. Nuclei were counterstained with DAPI (Beyotime, China). A fluorescence microscope was used to observe the sections (Olympus, Japan).

### 2.21 Statistical Analysis

Statistical analysis was conducted in SPSS v21.0 (SPSS, USA), and visualized by GraphPad Prism 8.0 (GraphPad, USA). All experiments were performed in triplicate, and results are presented as mean ± SD. For comparisons between two groups, Student's *t*‐test was applied when data met the assumptions of normality and homogeneity of variance; otherwise, the Mann–Whitney U test was used. For comparisons involving three or more groups, one‐way analysis of variance (ANOVA) followed by Tukey's post hoc test was conducted when data met both normality and homoscedasticity criteria. When these assumptions were violated, the Kruskal–Wallis test was employed, with Dunn's post hoc test applied for multiple comparison correction, with *p* < 0.05 considered statistically significant.

## 3 Results

### 3.1 Olfml3 Is Lowly Expressed in OSA and Suppresses Microglial Activation

**FIGURE 1:**
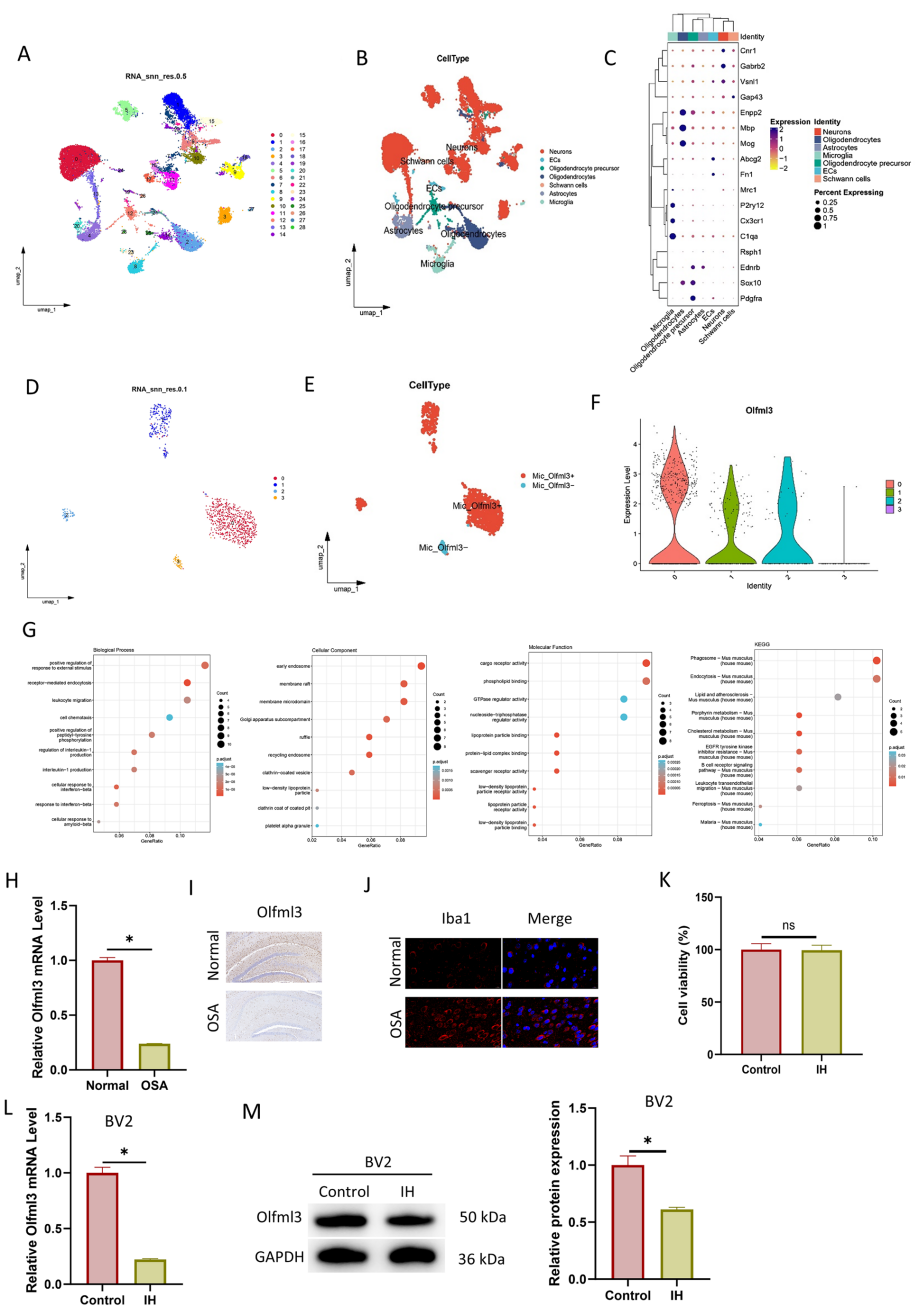
Olfml3 is Lowly Expressed in OSA and Suppresses Microglial Activation. (A) UMAP plot of annotated cell clusters; (B) UMAP plot of annotated cell types; (C) Marker genes used for cell type annotation; (D) UMAP plot of microglial clusters; (E) UMAP plot of microglial clusters; (F) Expression of Olfml3 in microglial clusters; (G) GO and KEGG enrichment analyses of cluster 3; (H) QRT‐PCR analysis of Olfml3 mRNA levels in Normal and OSA samples; (I) IHC detection of Olfml3 protein levels in Normal and OSA samples; (J) IF detection of Iba1 expression in Normal and OSA samples; (K) Cell viability assessed by CCK‐8 assay in Control and IH‐treated cells; (L) QRT‐PCR analysis of Olfml3 mRNA levels in Control and IH‐treated cells; (M) WB detection of Olfml3 protein levels (left) and corresponding quantification (right) in Control and IH‐treated cells. *p < 0.05.

scRNA‐seq data were collected from normoxic and hypoxic mouse samples in the SRA database (PRJNA1089719). After preprocessing the dataset, we obtained 20,376 cells and 28,158 genes. Using UMAP visualization, the cells were divided into 29 major clusters (Figure 1A). These clusters were annotated based on corresponding cell type markers, and the UMAP plots of each cell type in the samples are shown in Figure 1B,C. The cell types included neurons, endothelial cells (ECs), oligodendrocyte precursor cells, oligodendrocytes, Schwann cells, astrocytes, and microglia. Activated microglia are closely associated with the initiation and maintenance of neuroinflammation in OSA patients [6]. Based on this, we extracted microglial cells from the data and performed cluster analysis, identifying four distinct clusters (Figure 1D). By incorporating the target gene into the analysis, it was found that Olfml3 was highly expressed in clusters 0, 1, and 2 but exhibited low expression in cluster 3 (Figure 1E,F). Enrichment analysis performed on clusters 0, 1, and 2 revealed that the Mic_Olfml3+ subcluster was enriched in pathways associated with promoting long‐term neuronal survival and functional recovery (Figure S1A–C). Further GO‐KEGG enrichment analyses of cluster 3, which had low Olfml3 expression, revealed enrichment in pathways such as monocyte proliferation, leukocyte proliferation, EC migration, IL‐6 production, and microglial development and activation (Figure 1G). The activation of these pathways synergistically enhances various aspects of inflammatory responses, including increasing immune cell numbers and activity, altering vascular ECs, and the production and release of critical inflammatory mediators, thus exacerbating inflammation [17, 18].

To further validate the expression of Olfml3 under IH, we established an OSA animal model induced by IH. qRT‐PCR and IHC assays demonstrated that Olfml3 levels were significantly reduced in OSA (Figure 1H,I). Then, IF was used to detect the expression of Iba1, a marker of microglial activation, in mouse neuronal tissues. The results showed that microglial activation was suppressed in the OSA model compared to the control group (Figure 1 J). Finally, we constructed an IH‐induced model using BV2 cells. CCK‐8 assay confirmed that IH exposure did not induce overt nonspecific cell death (Figure 1 K). Subsequently, qRT‐PCR and WB analysis confirmed that IH induction significantly decreased Olfml3 expression in vitro (Figure 1 L‐M). In conclusion, these results indicated that Olfml3 is lowly expressed in OSA and suppresses microglial activation.

### 3.2 Olfml3 Overexpression Attenuates Microglial Inflammation and Suppresses Neuronal Injury

**FIGURE 2:**
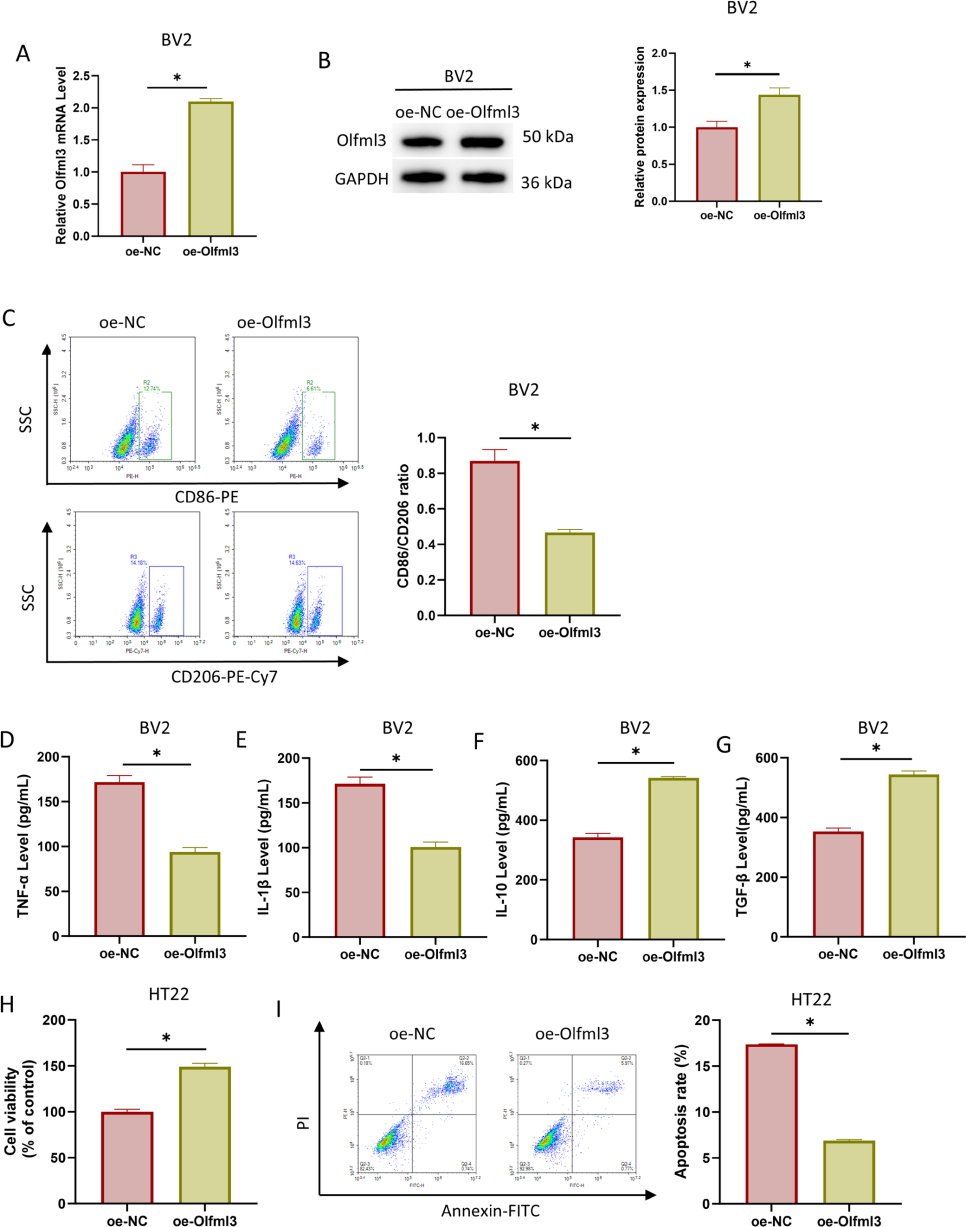
Olfml3 Overexpression Attenuates IH‐Induced Pro‐Inflammatory Microglial Activation and Suppresses Neuronal Injury. Cell groups: Oe‐NC, oe‐Olfml3. (A) QRT‐PCR analysis of Olfml3 mRNA levels; (B) WB detection of Olfml3 protein levels (left) and corresponding quantification (right); (C) Flow cytometry analysis of BV2 M1/M2 polarization; (D–G) ELISA measurement of TNF‐α (D), IL‐1β (E), IL‐10 (F), and TGF‐β (G) in BV2 cells; (H) CCK‐8 assay assessing HT‐22 cell viability; (I) Flow cytometry analysis of HT‐22 apoptosis. *p < 0.05.

Previous analyses revealed that cluster 3, which exhibited low Olfml3 expression, was enriched in pathways that synergistically exacerbate inflammation. Therefore, we investigated whether Olfml3 influences microglial inflammation. First, we constructed BV2 cells overexpressing Olfml3 and confirmed successful transfection through qRT‐PCR and WB (Figure 2A,B). Flow cytometry analysis of the CD86+/CD206+ ratio demonstrated that Olfml3 overexpression significantly attenuated IH‐induced pro‐inflammatory microglial activation (Figure 2C). Additionally, ELISA was used to measure the levels of key inflammatory cytokines. The results showed that the Olfml3 upregulation in BV2 cells was significantly associated with the reduction of pro‐inflammatory factors and the increase of anti‐inflammatory factors, which further confirmed that Olfml3 suppressed microglial inflammation (Figure 2D–G). Next, we collected CM from different microglial groups and used it to culture HT‐22 neuronal cells. CCK‐8 assays revealed that CM from Olfml3‐overexpressing microglia significantly enhanced HT‐22 cell viability, while flow cytometry showed that it suppressed HT‐22 apoptosis (Figure 2H,I). Collectively, Olfml3 alleviates IH‐induced microglial inflammation and protects neurons from injury.

### 3.3 Olfml3 Suppresses the Cybb‐Mediated TLR4/NF‐κB Pathway in IH‐Treated Microglia

**FIGURE 3:**
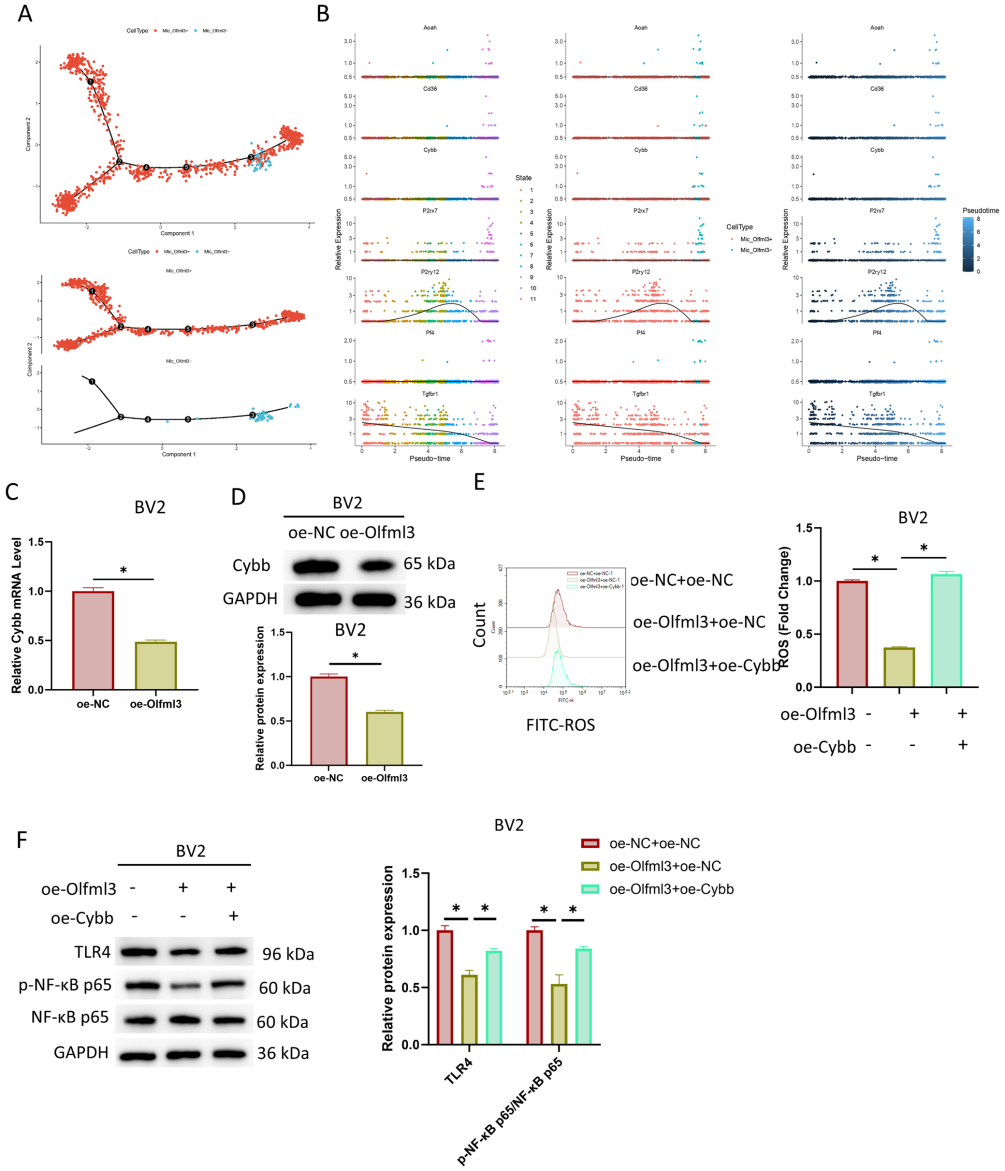
Olfml3 Blocks the Cybb‐Mediated TLR4/NF‐κB Pathway in Microglia Under IH Treatment. (A) Distribution of microglial subpopulations along the differentiation trajectory; (B) Gene expression dynamics during pseudotime progression. Cell groups (oe‐NC, oe‐Olfml3): (C) QRT‐PCR analysis of Cybb mRNA levels; (D) WB detection of Cybb protein levels (upper panel) and corresponding quantification (lower panel). Cell groups (oe‐NC + oe‐NC, oe‐Olfml3 + oe‐NC, oe‐Olfml3 + oe‐Cybb): (E) ROS levels in BV2 cells measured by fluorescent probe; (F) WB analysis of TLR4, p‐NF‐κB p65, and NF‐κB p65 expression (left) and corresponding quantification (right). *p < 0.05.

To further elucidate the differentiation mechanism of microglia, we performed pseudotime trajectory analysis on microglial subpopulations using Monocle 2. The results showed that Mic_Olfml3‐ were predominantly distributed at the late stage of differentiation, expanding along the trajectory (Figure 3A). Additionally, we examined gene expression changes during differentiation and found that Aoah, Cybb, and P2rx7 were significantly upregulated in Mic_Olfml3‐ (Figure 3B). Since Cybb is involved in ROS production and ROS can activate inflammatory pathways such as TLR4/NF‐κB [19], we hypothesized that Olfml3's neuroprotective effects might be linked to the Cybb‐mediated inflammatory pathway.

To validate this, we constructed BV2 cells with Olfml3 overexpression and assessed Cybb expression. qRT‐PCR and WB confirmed that Olfml3 overexpression downregulated Cybb, which demonstrated an inverse correlation between the two (Figure 3C,D). Next, we conducted a rescue experiment by co‐transfecting oe‐Olfml3 and oe‐Cybb into BV2 cells under IH conditions. Using a fluorescent probe, we found that Olfml3 overexpression significantly reduced ROS levels, while Cybb upregulation reversed this effect (Figure 3E). Furthermore, WB analysis revealed that Olfml3 overexpression suppressed TLR4 protein levels and NF‐κB phosphorylation, whereas Cybb overexpression reactivated the TLR4/NF‐κB pathway (Figure 3F). These findings indicated that Olfml3 suppresses Cybb expression in microglia under IH conditions, thereby suppressing the TLR4/NF‐κB inflammatory pathway.

### 3.4 Olfml3 Suppresses Microglial Inflammation and Neuronal Injury Through the Cybb/TLR4/NF‐κB Axis

**FIGURE 4:**
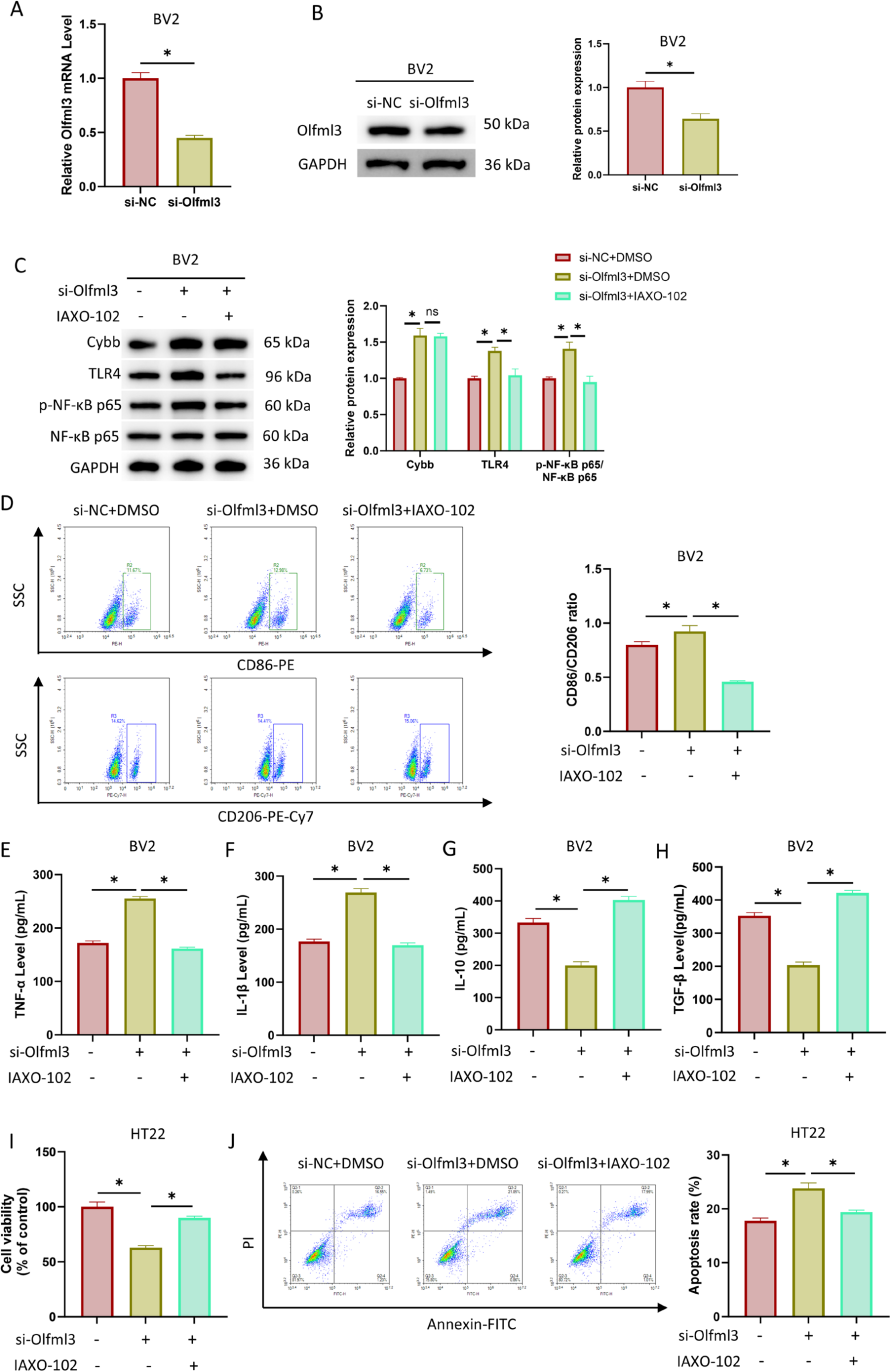
Olfml3 Ameliorates Microglial Inflammation and Neuronal Injury via the Cybb‐Mediated TLR4/NF‐κB Pathway. Cell treatment groups: si‐NC, si‐Olfml3: (A) QRT‐PCR analysis of Olfml3 mRNA levels; (B) WB detection of Olfml3 protein expression (left) and corresponding quantification (right). Cell groups: si‐NC + DMSO, si‐Olfml3 + DMSO, si‐Olfml3 + IAXO‐102: (C) WB analysis of Cybb, TLR4, p‐NF‐κB p65 and NF‐κB p65 expression in BV2 cells (left) and corresponding quantification (right); (D) Flow cytometry analysis of BV2 cell M1/M2 polarization; (E–H) ELISA detection of TNF‐α (E), IL‐1β (F), IL‐10 (G) and TGF‐β (H) levels in BV2 cells; (I) CCK‐8 assay measuring HT22 cell viability; (J) Flow cytometry analysis of HT22 cell apoptosis. *p < 0.05.

Research has demonstrated that activation of the TLR4/NF‐κB pathway triggers the release of inflammatory mediators (such as TNF‐α and IL‐1β), thereby exacerbating inflammatory responses. We therefore hypothesized that Olfml3's effects on microglial inflammation might be associated with Cybb‐mediated activation of this pathway. First, we performed Olfml3 knockdown, with successful transfection confirmed by qRT‐PCR and WB (Figure 4A,B). Rescue experiments were conducted by adding either DMSO or the TLR4/NF‐κB pathway inhibitor IAXO‐102 (MCE, USA). WB analysis revealed that Cybb expression was upregulated following Olfml3 knockdown, while IAXO‐102 treatment had no effect on Cybb levels. However, Olfml3 knockdown promoted activation of this pathway (Figure 4C). Subsequent experiments examining CD86/CD206 ratios and inflammatory cytokine expression demonstrated that Olfml3 knockdown exacerbated microglial inflammation. This inflammatory response was significantly ameliorated when the TLR4/NF‐κB pathway was inhibited (Figure 4D–H). Finally, we cultured neuronal cells with CM from differentially treated groups. The results showed that inhibition of the TLR4/NF‐κB pathway alleviated the neuronal injury caused by si‐Olfml3 treatment (Figure 4I,J). In summary, these findings demonstrated that Olfml3 suppresses microglial inflammation and neuronal injury through the Cybb/TLR4/NF‐κB axis.

### 3.5 Animal Experiments Validate That Olfml3 Suppresses Microglial Inflammation and Neuronal Injury Through the Cybb‐Mediated TLR4/NF‐κB Pathway

**FIGURE 5:**
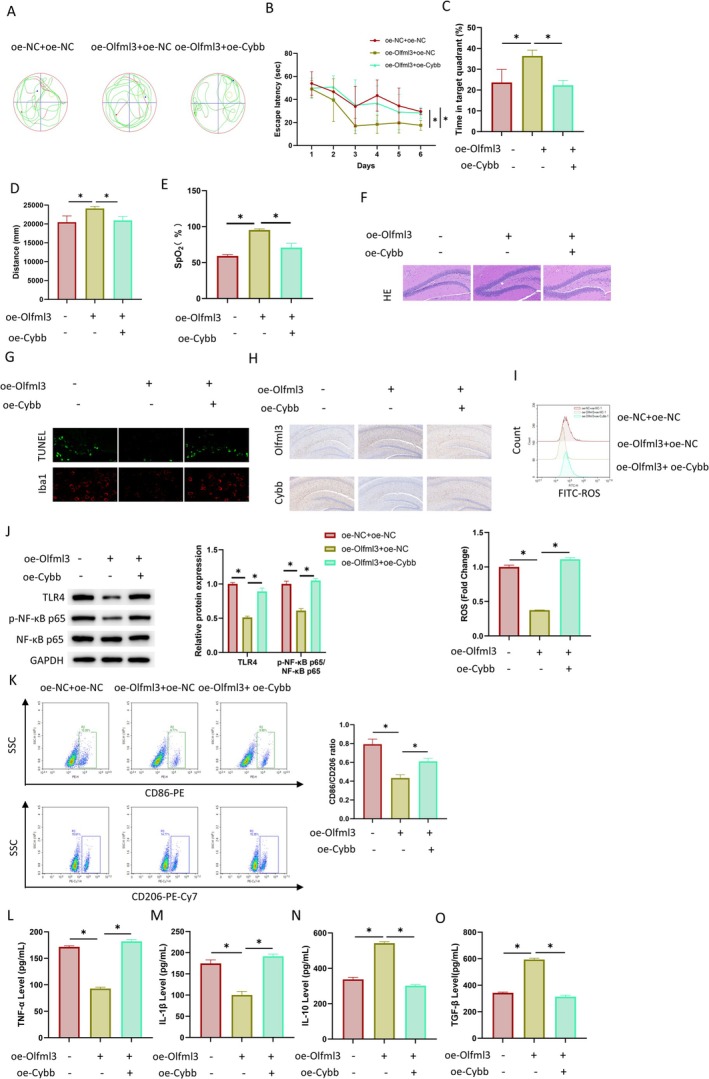
Animal Experiments Validate that Olfml3 Suppresses Microglial Inflammation and Neuronal Injury through the Cybb‐Mediated TLR4/NF‐κB Pathway. Animal groups: oe‐NC + oe‐NC, oe‐Olfml3 + oe‐NC, oe‐Olfml3 + oe‐Cybb. (A) MWM trajectory maps; (B) Average escape latency time; (C) Mean percentage of time spent in the target quadrant; (D) Platform crossing distance of mice; (E) Measurement of SpO_2_; (F) HE staining of neuronal tissue; (G) TUNEL assay for neuronal apoptosis and IF detection of Iba1 levels; (H) IHC detection of Olfml3 and Cybb expression; (I) ROS detection; (J) WB analysis of TLR4, p‐NF‐κB p65, and NF‐κB p65 expression (left) and corresponding quantification (right); (K) Flow cytometry analysis of BV2 cell M1/M2 polarization; (L–O) ELISA measurement of TNF‐α (L), IL‐1β (M), IL‐10 (N), and TGF‐β (O) levels in BV2 cells. *p < 0.05.

Finally, we established an IH mouse model to further validate the effect of Olfml3 on microglial inflammation and neuronal injury through the Cybb‐mediated TLR4/NF‐κB pathway. Lentiviruses carrying oe‐Olfml3 or oe‐Cybb were injected into the cerebral ventricles of mice according to the experimental groups, followed by IH treatment. The MWM test was conducted to assess cognitive dysfunction in mice. The results showed that compared with the control group, mice in the oe‐Olfml3 group exhibited reduced escape latency, increased percentage of time spent in the target quadrant, and increased time of platform crossings, indicating that Olfml3 overexpression alleviated deficits in learning and memory functions. In contrast, Cybb overexpression significantly reversed these improvements (Figure 5A–D). Subsequent measurements revealed that overexpression of Olfml3 resulted in a marked decrease in SpO_2_ levels in mice, an effect that was significantly reversed upon upregulation of Cybb (Figure 5E). Additionally, oe‐Cybb impaired the increase in neuronal volume and relatively normal morphology in the hippocampal region of Olfml3‐overexpressing mice (Figure 5F). TUNEL staining revealed fewer TUNEL‐positive cells upon Olfml3 upregulation, whereas neuronal apoptosis worsened after Cybb upregulation (Figure 5G). Furthermore, IF staining of Iba1 demonstrated that oe‐Olfml3 suppressed microglial activation, while Cybb upregulation increased it (Figure 5G). IHC detection of Olfml3 and Cybb levels further confirmed their negative correlation (Figure 5H). Assessments of ROS and the TLR4/NF‐κB pathway yielded results consistent with in vitro experiments, indicating that Olfml3 reduced ROS levels and suppressed the activation of this signaling pathway by regulating Cybb expression (Figure 5I,J). Finally, the ratios of CD86 and CD206 and the expression of inflammatory factors revealed that the ameliorative effect of Olfml3 on microglial inflammation was suppressed upon Cybb overexpression (Figure 5 K–O). In summary, these in vivo experiments further validated that Olfml3 suppresses microglial inflammation and neuronal injury via the Cybb‐mediated TLR4/NF‐κB pathway.

## 4 Discussion

One of the primary pathophysiological features of OSA is neuroinflammation, which is closely associated with microglial activation [6]. Research has reported that oxidative stress and inflammatory responses exert detrimental effects on microglia, inducing their apoptosis [20]. Meanwhile, impaired microglia can further amplify the inflammatory cascade by releasing large amounts of pro‐inflammatory factors [12]. Therefore, protecting microglia from inflammatory damage is a key strategy for mitigating neuroinflammation in OSA. Our study revealed that Olfml3 significantly suppresses microglial inflammation and alleviates neuronal injury through the Cybb/TLR4/NF‐κB axis, which provided a novel therapeutic direction for OSA‐associated neuroinflammation.

The research collected scRNA‐seq data of mouse hippocampal samples under normoxic and hypoxic conditions for analysis and identified 7 different cell types. Microglia, the immune cells of the CNS, may be activated and migrate to injury sites, or their numbers may decline due to dysfunction or apoptosis caused by chronic inflammation [21, 22]. Multiple studies have demonstrated that IH induces microglial activation, consequently leading to neuroinflammation and neuronal injury [23, 24], which was similarly confirmed by our OSA mouse model. Olfml3, a secreted glycoprotein, is predominantly expressed in microglia [25]. In our findings, Olfml3 was downregulated in IH‐treated OSA brain tissues and microglia, suggesting its potential involvement in the microglial activation mechanism. Of note, as a secreted protein, Olfml3 possesses an intrinsic advantage for serving as a biomarker in liquid biopsies—offering the potential to assess the severity of OSA‐related neuroinflammation or even estimate the risk of cognitive impairment by measuring its levels in patients' cerebrospinal fluid or peripheral blood. The precise role of Olfml3 in microglia neuroinflammation and its underlying regulatory mechanisms remains uncovered.

GO and KEGG enrichment analyses revealed that cluster 3 (with Olfml3 low expression) was enriched in pathways related to EC migration, IL‐6 production, and microglial development and activation. The activation of these pathways synergistically enhances various aspects of inflammation, including increased immune cell recruitment and activation, vascular endothelial alterations, and the production and release of key inflammatory mediators, thereby exacerbating inflammatory progression [26, 27]. The role of Olfml3 in neuroinflammation has been previously reported by Nicolas Neidert's study: Olfml3 expression in microglia is regulated by the TGFβ1/Smad2 pathway, which plays a crucial role in modulating immune responses and inflammation [25]. We found that Olfml3 promoted the microglial polarization shift from M1 to M2, suggesting its potential anti‐inflammatory effect via M1 phenotype suppression. Of note, the enrichment analyses of clusters 0, 1, and 2 revealed that Mic_Olfml3+ was enriched in pathways related to promoting the long‐term survival and functional recovery of neurons (Figure S1A–C), indicating a possible neuroprotective role of Mic_Olfml3 + [28, 29]. These findings provide a theoretical basis for therapeutic strategies targeting Olfml3: If its expression could be upregulated through pharmacological or genetic interventions, it might promote microglial polarization toward the M2 phenotype, thereby halting the cascade of neuroinflammation at an early stage of OSA and protecting neurons from injury. Microglia secrete inflammatory mediators and cytokines that contribute to neuronal injury [30]. Based on this, we collected CM from BV2 microglia to culture HT22 neurons and provided evidence for the first time that Olfml3 in microglia had protective effects on neurons. Nevertheless, the mechanisms by which Olfml3 regulates microglial inflammation and neuronal injury require further investigation.

Next, by analyzing gene expression changes during Olfml3 differentiation, we observed that Aoah, Cybb, and P2rx7 were markedly upregulated in Mic_Olfml3‐. Cybb is proven to be involved in ROS generation, which can activate inflammatory pathways such as TLR4/NF‐κB [19]. Wu et al. [31]. previously demonstrated that Cybb promotes ROS production and NF‐κB activation, and thus influences the inflammatory microenvironment and neuronal apoptosis. Building upon these insights, our study further advances the understanding by positioning Cybb downstream of Olfml3 and validating its role in the pathological context of OSA. Activation of the TLR4/NF‐κB pathway promotes the release of proinflammatory cytokines such as TNF‐α and IL‐1β, thereby exacerbating local inflammatory responses. Moreover, this pathway contributes to disease progression by inducing oxidative stress and damaging both the vascular and nervous systems [32]. Although the central role of the TLR4/NF‐κB axis in microglial inflammation and neuronal injury has been established across various central nervous system disorders, such as traumatic brain injury [33], spinal cord injury [34], and ischemic stroke [35]. However, no studies elucidated the specific mediating role of Cybb in the TLR4/NF‐κB pathway. Through in vitro and in vivo experiments, we confirmed that Olfml3 suppressed the TLR4/NF‐κB pathway through Cybb, thereby ameliorating microglial inflammation and neuronal injury in OSA. This finding not only underscores the pivotal role of Olfml3 in OSA but also identifies a defined molecular target for pharmacological intervention. As a core subunit of NADPH oxidase, Cybb is already the focus of various small‐molecule inhibitors in preclinical development aimed at modulating its activity or expression. Future investigations may seek to develop novel therapeutic strategies targeting the Olfml3/Cybb axis, offering a new avenue for the treatment of OSA‐associated neuroinflammation. However, although our findings demonstrate a negative correlation between Olfml3 and Cybb expression, and indicate that Olfml3 regulates the TLR4/NF‐κB pathway through Cybb, the precise molecular mechanism by which Olfml3—acting as a secreted glycoprotein—modulates Cybb expression remains elusive. Whether Olfml3 engages a putative receptor to activate downstream signaling cascades that indirectly govern Cybb transcription, or exerts its effects through alternative indirect mechanisms influencing Cybb mRNA stability or translational efficiency, is still unclear. This mechanistic gap represents the initial link in the proposed signaling pathway and will be a focus of our future investigations.

In summary, we demonstrated that Olfml3 suppresses microglial inflammation by shifting their polarization from the M1 to the M2 phenotype by integrating scRNA‐seq with in vivo and in vitro experiments. Microglial Olfml3 exerts anti‐inflammatory effects by inhibiting the Cybb‐mediated TLR4/NF‐κB pathway to mitigate neuronal injury. This study elucidates the neuroprotective role of Olfml3‐mediated microglial anti‐inflammatory polarization in OSA, offering novel perspectives on the pivotal function of microglial polarization in CNS disorders. Nevertheless, several limitations should be acknowledged in this study. First, the absence of validation using clinical samples restricts the translational potential of our findings. The current results are primarily derived from single‐cell RNA sequencing, along with animal and cellular experiments, and have yet to be corroborated in clinical specimens from patients with OSA. Future studies will aim to collect blood samples or cerebrospinal fluid from individuals with OSA to assess Olfml3 expression levels and evaluate their correlation with disease severity—such as the apnea–hypopnea index and minimum oxygen saturation—as well as with cognitive performance scores. Second, the precise mechanism by which Olfml3 regulates Cybb remains to be fully elucidated. Although an association between Olfml3 and Cybb expression has been established, whether this regulation occurs at the transcriptional level, via protein–protein interactions, or through other indirect pathways warrants further investigation. Subsequent studies integrating clinical samples with multi‐omics analyses, along with molecular approaches such as chromatin immunoprecipitation and co‐immunoprecipitation, will be essential to delineate the mechanistic basis of Olfml3‐mediated regulation of Cybb and to comprehensively explore its functional role in neuroinflammation‐associated diseases.

## Author Contributions

Conceptualization: Deqiu Kong, Yaowen Wang, Xianjun Chen, Cihao Hu, Bin Zhang, Xing Chen. Methodology: Deqiu Kong, Yaowen Wang. Formal analysis and investigation: Xianjun Chen, Cihao Hu, Bin Zhang, Xing Chen. Writing – original draft preparation: Deqiu Kong, Yaowen Wang, Xianjun Chen. Writing – review and editing: Deqiu Kong, Yaowen Wang, Xianjun Chen, Cihao Hu. Funding acquisition: Deqiu Kong, Yaowen Wang. Resources: Bin Zhang. Supervision: Xing Chen.

## Funding

Zhejiang Provincial Medical and Health Science and Technology Program, 2025KY1326. Zhejiang Provincial Traditional Chinese Medicine Science and Technology Program, 2024ZL896.

## Ethics Statement

Experiments were performed under a project license (No. D202506‐14) granted by the ethics committee of the Ethics Committee for Animal Experiments of Guangdong Medical Laboratory Animal Center, in compliance with institutional guidelines for the care and use of animals.

## Consent

This study did not involve human participants or personal data collection. Therefore, informed consent was not applicable.

## Conflicts of Interest

The authors declare no conflicts of interest.

## Supporting information


**Figure S1:** Enrichment Analyses of Mic_Olfml3+ Pathways: (A) GO and KEGG enrichment analyses of cluster 0; (B) GO and KEGG enrichment analyses of cluster 1; (C) GO and KEGG enrichment analyses of cluster 2.

**Figure S2:** Schematic Diagram of Transfection Efficiency Validation.

**Supporting Information: 1** siRNA sequence information of Olfml3, Full‐length CDS sequence of Olfml3, and Cybb.

## Data Availability

The data that support the findings of this study are available from the corresponding author upon reasonable request.
