# Supplementary figures and images for: Olfml3 Regulates Microglial Inflammation and Neuronal Injury in Obstructive Sleep Apnea via Cybb‐Mediated TLR4/NF‐κB Pathway
Source: CNS Neurosci Ther. 2026 Jul 8;32(7):e71006. doi: 10.1002/cns.71006 (PMC13344635; doi:10.1002/cns.71006)

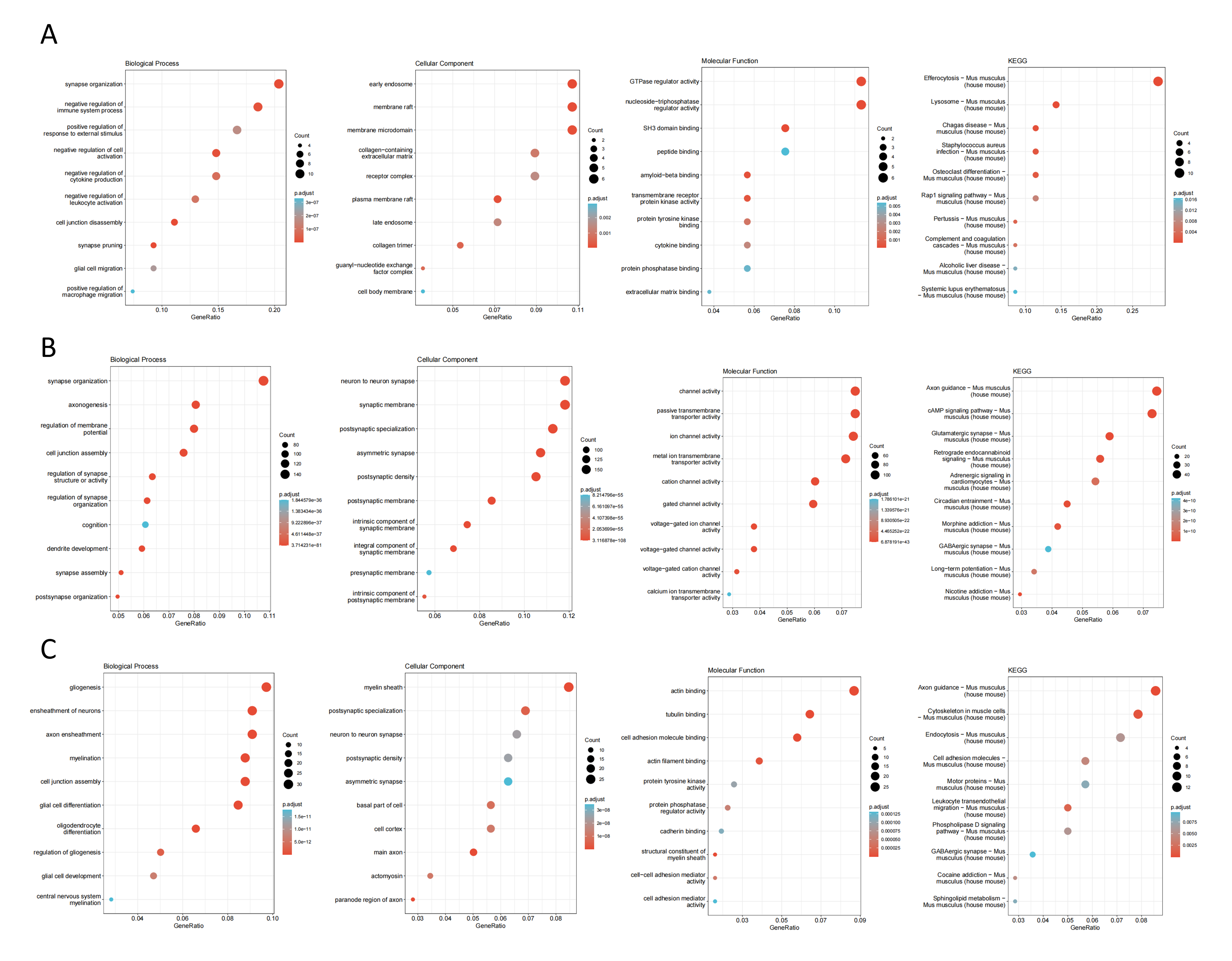

Supplement: Supplementary file 1 — Figure S1: Enrichment Analyses of Mic_Olfml3+ Pathways: (A) GO and KEGG enrichment analyses of cluster 0; (B) GO and KEGG enrichment analyses of cluster 1; (C) GO and KEGG enrichment analyses of cluster 2. [file CNS-32-e71006-s002.tif]

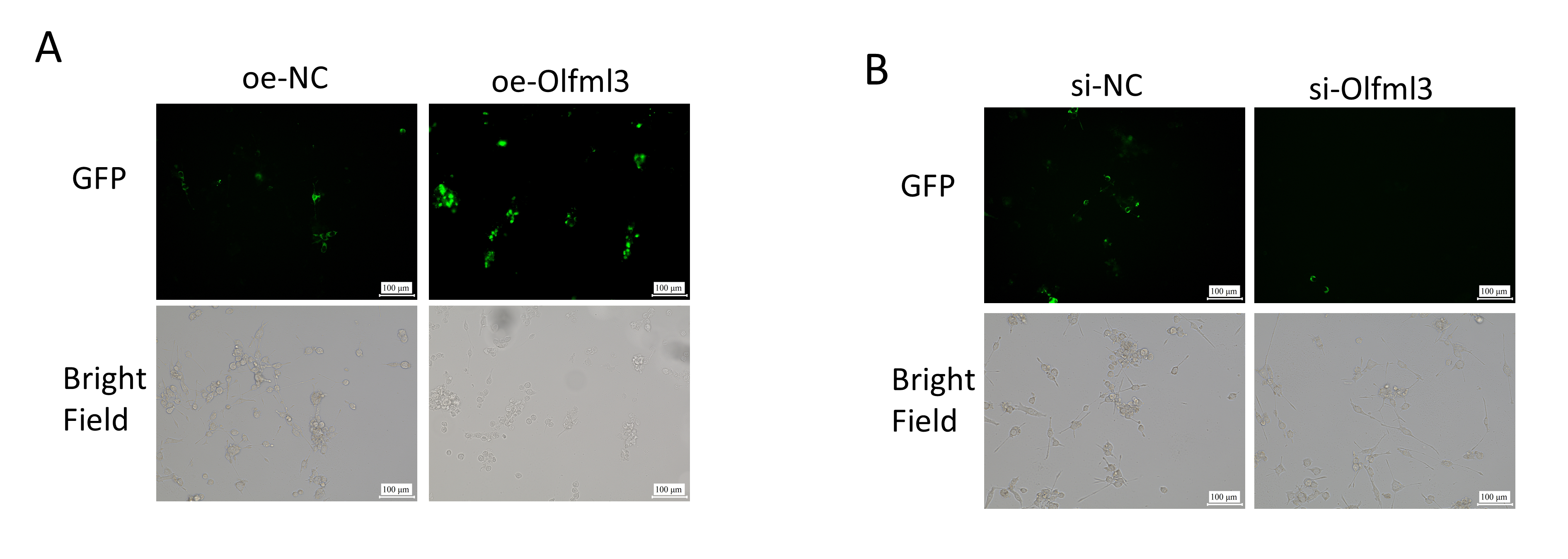

Supplement: Supplementary file 2 — Figure S2: Schematic Diagram of Transfection Efficiency Validation. [file CNS-32-e71006-s003.tif]
