# Supplementary material for: Olfml3 Regulates Microglial Inflammation and Neuronal Injury in Obstructive Sleep Apnea via Cybb‐Mediated TLR4/NF‐κB Pathway
Source: CNS Neurosci Ther. 2026 Jul 8;32(7):e71006. doi: 10.1002/cns.71006 (PMC13344635; doi:10.1002/cns.71006)
Supplement: Supplementary file 3 — Supporting Information: 1 siRNA sequence information of Olfml3, Full‐length CDS sequence of Olfml3, and Cybb. [file CNS-32-e71006-s001.docx]

**Supplementary Material 1**

siRNA sequence information of Olfml3, Full-length CDS sequence of Olfml3 and Cybb.

siRNA sequence information of Olfml3

| Olfml3-siRNA1 | GAUUGUCUAUAAGCUGGAGAU |
| --- | --- |
| Olfml3-siRNA2 | AUCUCCAGCUUAUAGACAAUC |

Full-length CDS sequence of Olfml3

1 gactgtacgt tccttctact ctggcaccac tctccaggct gccatggggc ccagcacccc

61 tctcctcatc ttgttccttt tgtcatggtc gggacccctc caaggacagc agcaccacct

121 tgtggagtac atggaacgcc gactagctgc tttagaggtt gagatccgta gcattttagt

181 gtccgagagg ataattgcta agggagcaga gacctcttca caccttcatg cgcttagacc

241 tgcctagagg ctcagatggc tcagtggcac tgctcacaga gaacacaggc tggaggacat

301 ggagtcgggg aacggctggc ccagtgccag gaccagagta gtcggcatgc tgctgagctg

361 cgggacttca agaacaagat gctgccactg ctggaggtgg cagagaagga gcgggaggca

421 ctcagaactg aggccgacac catctccggg agagtggatc gtctggagcg ggaggtagac

481 tatctggaga cccagaaccc agctctgccc tgtgtagagt ttgatgagaa ggtgactgga

541 ggccctggga ccaaaggcaa gggaagaagg aatgagaagt acgatatggt gacagactgt

601 ggctacacaa tctctcaagt gagatcaatg aagattctga agcgatttgg tggcccagct

661 ggtctatgga ccaaggatcc actggggcaa acagagaaga tctacgtgtt agatgggaca

721 cagaatgaca cagcctttgt cttcccaagg ctgcgtgact tcacccttgc catggctgcc

781 cggaaagctt cccgagtccg ggtgcccttc ccctgggtag gcacagggca gctggtatat

841 ggtggctttc tttattttgc tcggaggcct cctggaagac ctggtggagg tggtgagatg

901 gagaacactt tgcagctaat caaattccac ctggcaaacc gaacagtggt ggacagctca

961 gtattcccag cagaggggct gatccccccc tacggcttga cagcagacac ctacatcgac

1021 ctggcagctg atgaggaagg tctttgggct gtctatgcca cccgggagga tgacaggcac

1081 ttgtgtctgg ccaagttaga tccacagaca ctggacacag agcagcagtg ggacacacca

1141 tgtcccagag agaatgctga ggctgccttt gtcatctgtg ggaccctcta tgtcgtctat

1201 aacacccgtc ctgccagtcg ggcccgcatc cagtgctcct ttgatgccag cggcaccctg

1261 acccctgaac gggcagcact cccttatttt ccccgcagat atggtgccca tgccagcctc

1321 cgctataacc cccgagaacg ccagctctat gcctgggatg atggctacca gattgtctat

1381 aagctggaga tgaggaagaa agaggaggag gtttgaggag ctagccttgt tttttgcatc

1441 tttctcactc ccatacattt atattatatc cccactaaat ttcttgttcc tcattcttca

1501 aatgtgggcc agttgtggct caaatcctct atatttttag ccaatggcaa tcaaattctt

1561 tcagctcctt tgtttcatac ggaactccag atcctgagta atccttttag agcccgaaga

1621 gtcaaaaccc tcaatgttcc ctcctgctct cctgccccat gtcaacaaat ttcaggctaa

1681 ggatgcccca gacccagggc tctaaccttg tatgcgggca ggcccaggga gcaggcagca

1741 gtgttcttcc cctcagagtg acttggggag ggagaaatag gaggagacgt ccagctctgt

1801 cctctcttcc tcactcctcc cttcagtgtc ctgaggaaca ggactttctc cacattgttt

1861 tgtattgcaa cattttgcat taaaaggaaa atccactgct

Full-length CDS sequence of Cybb

1 acattcaacc tctgccacca tggggaactg ggctgtgaat gaggggctct ccatttttgt

61 cattctggtt tggctggggt tgaacgtctt cctctttgtc tggtattacc gggtttatga

121 tattccacct aagttctttt acacaagaaa acttcttggg tcagcactgg cactggccag

181 ggcccctgca gcctgcctga atttcaactg catgctgatt ctcttgccag tctgtcgaaa

241 tctgctgtcc ttcctcaggg gttccagtgc gtgctgctca acaagagttc gaagacaact

301 ggacaggaat ctcacctttc ataaaatggt ggcatggatg attgcacttc actctgcgat

361 tcacaccatt gcacatctat ttaatgtgga atggtgtgtg aatgcccgag tcaataattc

421 tgatccttat tcagtagcac tctctgaact tggagacagg caaaatgaaa gttatctcaa

481 ttttgctcga aagagaataa agaaccctga aggaggcctg tacctggctg tgaccctgtt

541 ggcaggcatc actggagttg tcatcacgct gtgcctcata ttaattatca cttcctccac

601 caaaaccatc cggaggtctt actttgaagt cttttggtac acacatcatc tctttgtgat

661 cttcttcatt ggccttgcca tccatggagc tgaacgaatt gtacgtgggc agaccgcaga

721 gagtttggct gtgcataata taacagtttg tgaacaaaaa atctcagaat ggggaaaaat

781 aaaggaatgc ccaatccctc agtttgctgg aaaccctcct atgacttgga aatggatagt

841 gggtcccatg tttctgtatc tctgtgagag gttggtgcgg ttttggcgat ctcaacagaa

901 ggtggtcatc accaaggtgg tcactcaccc tttcaaaacc atcgagctac agatgaagaa

961 gaaggggttc aaaatggaag tgggacaata catttttgtc aagtgcccaa aggtgtccaa

1021 gctggagtgg caccctttta cactgacatc cgcccctgag gaagacttct ttagtatcca

1081 tatccgcatc gttggggact ggacagaggg gctgttcaat gcttgtggct gtgataagca

1141 ggagtttcaa gatgcgtgga aactacctaa gatagcggtt gatgggccct ttggcactgc

1201 cagtgaagat gtgttcagct atgaggtggt gatgttagtg ggagcaggga ttggggtcac

1261 acccttcgca tccattctca agtcagtctg gtacaaatat tgcaataacg ccaccaatct

1321 gaagctcaaa aagatctact tctactggct gtgccgggac acacatgcct ttgagtggtt

1381 tgcagatctg ctgcaactgc tggagagcca gatgcaggaa aggaacaatg ccggcttcct

1441 cagctacaac atctacctca ctggctggga tgagtctcag gccaatcact ttgctgtgca

1501 ccatgatgag gagaaagatg tgatcacagg cctgaaacaa aagactttgt atggacggcc

1561 caactgggat aatgaattca agacaattgc aagtcaacac cctaatacca gaataggagt

1621 tttcctctgt ggacctgaag ccttggctga aaccctgagt aaacaaagca tctccaactc

1681 tgagtctggc cctcggggag tgcatttcat tttcaacaag gaaaacttct aacttgtctc

1741 ttccatgagg aaataaatgt gggttgtgct gccaaatgct caaataatgc taattgataa

1801 tataaatacc ccctgcttaa aaatggacaa aaagaaacta taatgtaatg gttttccctt

1861 aaaggaatgt caaagattgt ttgatagtga taagttacat ttatgtggag ctctatggtt

1921 ttgagagcac ttttacaaac attatttcat ttttttcctc tcagtaatgt cagtggaagt

1981 tagggaaaag attcttggac tcaattttag aatcaaaagg gaaaggatca aaaggttcag

2041 taacttccct aagattatga aactgtgacc agatctagcc catcttactc caggtttgat

2101 actctttcca caatactgag ctgcctcaga atcctcaaaa tcagttttta tattccccaa

2161 aagaagaagg aaaccaagga gtagctatat atttctactt tgtgtcattt ttgccatcat

2221 tattatcata ctgaaggaaa ttttccagat cattaggaca taatacatgt tgagagtgtc

2281 tcaacactta ttagtgacag tattgacatc tgagcatact ccagtttact aatacagcag

2341 ggtaactggg ccagatgttc tttctacaga agaatattgg attgattgga gttaatgtaa

2401 tactcatcat ttaccactgt gcttggcaga gagcggatac tcaagtaagt tttgttaaat

2461 gaatgaatga atttagaacc acacaatgcc aagatagaat taatttaaag ccttaaacaa

2521 aatttatcta aagaaataac ttctattact gtcatagacc aaaggaatct gattctccct

2581 agggtcaaga acaggctaag gatactaacc aataggattg cctgaagggt tctgcacatt

2641 cttatttgaa gcatgaaaaa agagggttgg aggtggagaa ttaacctcct gccatgactc

2701 tggctcatct agtcctgctc cttgtgctat aaaataaatg cagactaatt tcctgcccaa

2761 agtggtcttc tccagctagc ccttatgaat attgaactta ggaattgtga caaatatgta

2821 tctgatatgg tcatttgttt taaataacac ccacccctta ttttccgtaa atacacacac

2881 aaaatggatc gcatctgtgt gactaatggt ttatttgtat tatatcatca tcatcatcct

2941 aaaattaaca acccagaaac aaaaatctct atacagagat caaattcaca ctcaatagta

3001 tgttctgaat atatgttcaa gagagagtct ctaaatcact gttagtgtgg ccaagagcag

3061 ggttttcttt ttgttcttag aactgctccc atttctggga actaaaacca gttttatttg

3121 ccccacccct tggagccaca aatgtttaga actcttcaac ttcggtaatg aggaagaagg

3181 agaaagagct gggggaaggg cagaagactg gtttaggagg aaaaggaaat aaggagaaaa

3241 gagaatggga gagtgagaga aaataaaaaa ggcaaaaggg agagagaggg gaagggggtc

3301 tcatattggt cattccctgc cccagatttc ttaaagtttg atatgtatag aatataattg

3361 aaggaggtat acacatattg atgttgtttt gattatctat ggtattgaat cttttaaaat

3421 ctggtcacaa attttgatgc tgagggggat tattcaaggg actaggatga actaaataag

3481 aactcagttg ttctttgtca tactactatt cctttcgtct cccagaatcc tcagggcact

3541 gagggtaggt ctgacaaata aggcctgctg tgcgaatata gcctttctga aatgtaccag

3601 gatggtttct gcttagagac acttaggtcc agcctgttca cactgcacct caggtatcaa

3661 ttcatctatt caacagatat ttattgtgtt attactatga gtcaggctct gtttattgtt

3721 tcaattcttt acaccaaagt atgaactgga gagggtacct cagttataag gagtctgaga

3781 atattggccc tttctaacct atgtgcataa ttaaaaccag cttcatttgt tgctccgaga

3841 gtgtttctcc aaggttttct atcttcaaaa ccaactaagt tatgaaagta gagagatctg

3901 ccctgtgtta tccagttatg agataaaaaa tgaatataag agtgcttgtc attataaaag

3961 tttccttttt tattctctca agccaccagc tgccagccac cagcagccag ctgccagcct

4021 agcttttttt tttttttttt ttttttagca cttagtattt agcatttatt aacaggtact

4081 ctaagaatga tgaagcattg tttttaatct taagactatg aaggtttttc ttagttcttc

4141 tgcttttgca attgtgtttg tgaaatttga atacttgcag gctttgtatg tgaataattc

4201 tagcggggga cctgggagat aattcctacg gggaattctt aaaactgtgc tcaactatta

4261 aaatgaatga gctttc
